# Re-Audit of Benzodiazepine and Z-Drug Prescribing in Two Community Treatment Teams in the Cumbria, Northumberland, Tyne and Wear Trust

**DOI:** 10.1192/bjo.2022.472

**Published:** 2022-06-20

**Authors:** Michelle Obayi, Helen Hargreaves, Niraj Ahuja

**Affiliations:** Cumbria, Northumberland, Tyne and Wear NHS Foundation Trust, Newcastle Upon Tyne, United Kingdom

## Abstract

**Aims:**

This is a re-audit of Benzodiazepine and Z-hypnotic drugs prescriptions in two community treatment teams (CTTs) in the Cumbria Northumberland Tyne and Wear (CNTW) Trust, comparing with previous audits in 2009, 2017 and 2018 to check whether areas of good practice were maintained, and progress was made.

**Methods:**

We reviewed caseloads of four CNTW consultants in the two CTTs which included 554 patient encounters with 60 encounters where benzodiazepines or z-drugs were prescribed. Nine missing data sets brought the total audit sample to 51. For these 51, prescribing information was gathered from RiO and assessed against standards derived from CNTW Trust Policy and BNF prescribing guidance. To be compliant, 90–100% of prescriptions needed to meet the standard.

**Results:**

Overall, the rate of prescribing of benzodiazepines and Z- drugs increased from previous audit (7% in 2018, now 10.8%). Good areas of practice maintained were as follows (all 100%): all teams were compliant in prescribing within BNF limits, refrained from prescribing diazepam in 10 mg formulation, and no pregnant/post-partum women were prescribed these medications.

Although non-compliant, there were clear improvements in documenting indicated use (2018: 61.65%, 2021: 80.8%), and providing prescriptions of <4 weeks in duration (2018: 58.2%, 2021: 79.2%)

Key areas of concern were as follows: poor documentation of indication, duration of treatment and plans for review/discontinuation (compliance ranged from 31.5% - 81.2% in these areas). There was poor documentation of what verbal advice was given (0–16.9%), and lack of clearly documented tapering/discontinuation plans for those on long-term prescriptions (16.1%). The provision of written advice reduced from previous audit (2018: 10.7%, 2021: 5.8%). As 41/51 encounters were via telephone or video due to COVID-19 pandemic, this may have impacted on results.

**Conclusion:**

Despite improvement in some areas, there remains scope for ongoing improvement in other areas. To improve these, we plan to produce and distribute an educational email to all prescribers, including the following: information on this audit and its findings, prescribing guidelines, relevant e-links to patient information leaflets as well as the audit proforma used for this audit, to encourage prescribers to undertake self-directed practice. A poster will be distributed, highlighting prescribing guidelines and standards, to be printed and displayed in clinical areas as reminder of prescribing responsibilities and the importance of documentation. Prescribers will be encouraged to participate in a small quiz to test learning. Efficacy of these measures will be assessed with a re-audit in one years’ time.

